# The gut microbiota modifies antibody durability and booster responses after SARS-CoV-2 vaccination

**DOI:** 10.1186/s12967-024-05637-2

**Published:** 2024-09-06

**Authors:** Hye Seong, Jin Gu Yoon, Eliel Nham, Yu Jung Choi, Ji Yun Noh, Hee Jin Cheong, Woo Joo Kim, Eui Ho Kim, Chulwoo Kim, Young-Hee Han, Sooyeon Lim, Joon Young Song

**Affiliations:** 1 Department of Internal Medicine, Guro Hospital, Korea University College of Medicine, Gurodong-Ro 148, Guro-Gu, Seoul, 08308 Republic of Korea; 2 Asia Pacific Influenza Institute, Guro Hospital, Korea University College of Medicine, Gurodong-Ro 148, Guro-Gu, Seoul, 08308 Republic of Korea; 3 Vaccine Innovation Center-KU Medicine, Seoul, Republic of Korea; 4 Viral Immunology Laboratory, Institut Pasteur Korea, Seongnam, Republic of Korea; 5 Department of Microbiology, Korea University College of Medicine, Seoul, Republic of Korea; 6 Department of Food and Nutrition, Chungbuk National University, Cheongju, Republic of Korea

**Keywords:** Gut Microbiome, Vaccination, SARS-CoV-2, Half-life, Immunogenicity

## Abstract

**Background:**

Severe acute respiratory syndrome coronavirus 2 (SARS-CoV-2) vaccines are pivotal in combating coronavirus disease 2019 (COVID-19); however, the declining antibody titers postvaccination pose challenges for sustained protection and herd immunity. Although gut microbiome is reported to affect the early antibody response after vaccination, its impact on the longevity of vaccine-induced antibodies remains unexplored.

**Methods:**

A prospective cohort study was conducted involving 44 healthy adults who received two doses of either the BNT162b2 or ChAdOx1 vaccine, followed by a BNT162b2 booster at six months. The gut microbiome was serially analyzed using 16S rRNA and shotgun sequencing, while humoral immune response was assessed using a SARS-CoV-2 spike protein immunoassay.

**Results:**

*Faecalibacterium prausnitzii* was associated with robust and persistent antibody responses post-BNT162b2 vaccination. In comparison, *Escherichia coli* was associated with a slower antibody decay following ChAdOx1 vaccination. The booster immune response was correlated with metabolic pathways involving cellular functions and aromatic amino acid synthesis.

**Conclusions:**

The findings of this study underscored the potential interaction between the gut microbiome and the longevity/boosting effect of antibodies following vaccination against SARS-CoV-2. The identification of specific microbial associations suggests the prospect of microbiome-based strategies for enhancing vaccine efficacy.

**Supplementary Information:**

The online version contains supplementary material available at 10.1186/s12967-024-05637-2.

## introduction

The global response to the coronavirus disease 2019 (COVID-19) pandemic has resulted in the development of new platform vaccines, including adenovirus vector, protein subunit, and mRNA vaccines, targeting severe acute respiratory syndrome coronavirus 2 (SARS-CoV-2). Although variable by vaccine platforms, SARS-CoV-2 vaccines elicited a robust antibody response, reaching peak titers at approximately 3–4 weeks after the second dose [1–4]. However, the antibody titers gradually decline over time, reaching levels similar to those of the initial immune response after approximately 6 months [5].

Post-vaccination antibody longevity plays a critical role in sustaining individual protection and establishing herd immunity. Long-lived plasma cells originating from naïve or memory B-cells are important for maintaining antibody longevity, with memory B-cells further enabling the quick and efficient response of the immune system when re-exposed to the relevant viral strains [6–8]. Recent studies have suggested a relatively consistent presence of SARS-CoV-2 vaccine-induced memory B cells for a 3–6 month period after vaccination, but their levels may vary among individuals [9, 10].

Immune responses following vaccination vary among individuals owing to the influence of several factors [11, 12], including the gut microbiota. The microbiota plays a central role in the host immune system, particularly in the training and maturation of key components of the adaptive immune system [13, 14]. First, gut microbes stimulate and shape the adaptive immune system through exposure to their bacterial components and active metabolites [15]. Second, the gut microbiota directly influences and modulates adaptive immunity [16]. The sustained interaction and maintenance of the complex balance between the gut microbiota and adaptive immune system are essential for intestinal homeostasis and the inhibition of inflammation, ensuring an optimal immune response and long-term protection after vaccination [17].

The relationship between peak antibody titers and the gut microbiota after vaccination against SARS-CoV-2 has been investigated in several studies [18–20]. However, the relationship between the durability of vaccine-induced antibodies and microbiota has not yet been studied. In this study, we investigated the association between the gut microbiota and durability of humoral immunity, which may provide insights into approaches for enhancing vaccine efficacy.

## Materials and methods

### Study design and participants

In this study, we prospectively recruited healthy adults who had received a single dose of the BNT162b2 or ChAdOx1 vaccines and were scheduled to get the second dose of the primary vaccination series. After enrollment, the participants were divided into two cohorts: those receiving two doses of the BNT162b2 vaccine (BNT162b2 cohort) and those receiving two doses of the ChAdOx1 vaccine (ChAdOx1 cohort). Following this, all participants were administered a booster vaccination with the BNT162b2 vaccine. Fecal and blood samples were collected at four time points: V1 (prior to the second dose of the primary vaccination series), V2 (3 weeks after the second dose), V3 (6 months after the second dose and prior to the booster dose), and V4 (3 weeks after the BNT162b2 booster dose). Data on patient demographics, medications, probiotics, supplements, laboratory results, and dietary records were collected at each time point.

Participants were excluded if they received medications that could have affected the gut microbiota, including antibiotics, laxatives, and motility drugs in the month prior to vaccination; had a history of positive SARS-CoV-2 results by nasopharyngeal PCR testing; or tested positive for serum antinuclear capsid protein (N) IgG. None of the participants contracted COVID-19 during the study period, as confirmed by anti-N IgG antibody testing (Abbott Laboratories, Chicago, IL, USA).

Fecal samples were collected in a nucleic acid preservation medium using Fecal Swab DNA Preservation and Transport Kits (Noble Bio, Hwaseong, South Korea) and stored at − 80 °C. Blood samples were collected in serum separation tubes via venipuncture. After centrifugation at 2500 rpm and − 4 °C for 10 min, the serum was transferred to clean plastic screw-capped vials and stored at − 80 °C.

The study protocol was approved by the Institutional Review Board of Korea University Guro Hospital (2021GR0097). Informed consent was obtained from all participants. All procedures were performed in accordance with the ethical standards of the relevant institutional and/or national research committees, the Helsinki Declaration of 1964 and its subsequent amendments, or comparable ethical standards.

### Classification of study groups based on the immune response after vaccination

Humoral immune responses were assessed using corresponding serum samples. Anti-S titers were measured using the Elecsys^®^ Anti-SARS-CoV-2 S assay kit (Roche, Rotkreuz, Switzerland) according to the manufacturer's protocol. Titers below the lower limit of quantitation were set to 0.4 U/mL.

The half-life of anti-S antibodies was determined from the V2–V3 time points. To investigate the microbial factors associated with the durability of vaccine-induced antibodies, participants from the BNT162b2 and ChAdOx1 vaccine cohorts were divided into slow- and fast-decay groups based on the median values of antibody half-life.

After receiving the BNT162b2 booster, the acute antibody responses were evaluated by comparing the fold changes in antibody titers between V3 and V4. Using the median fold-change in antibody titers across all booster vaccine recipients, participants were classified as either high or low responders.

### Analysis pipeline for 16S rRNA

Total DNA was extracted using the FastDNA^®^ SPIN Kit for Soil (MP Biomedicals, Santa Ana, CA, USA) according to the manufacturer's instructions. The extracted DNA was PCR amplified by CJ Bioscience (Seoul, Republic of Korea) using fusion primers targeting the V3–V4 regions of the 16S rRNA gene. PCR products were confirmed by electrophoresis on a 1% agarose gel, followed by visualization using the Gel Doc system (Bio-Rad, Hercules, CA, USA). The amplified products were purified using a CleanPCR kit (CleanNA, Waddinxveen, The Netherlands). Equal concentrations of purified products were pooled, and short fragments (nontarget products) were removed using a CleanPCR kit (CleanNA). The quality and size of products were assessed on a Bioanalyzer 2100 system (Agilent, Palo Alto, CA, USA) using a DNA 7500 chip. Mixed amplicons were pooled and sequenced using an Illumina MiSeq Sequencing System (Illumina, San Diego, CA, USA) according to the manufacturer's instructions. Analysis was performed using the EzBioCloud 16S-based Microbiome Taxonomic Profiling (MTP) and CJ Bioscience's bioinformatics cloud platform (https://www.ezbiocloud.net). All microbiome count data were normalized to that of 1000 read counts before further use.

The alpha diversity indices (abundance-based coverage estimator [ACE] and Simpson) were calculated as previously described [21–26]. To visualize sample differences, beta diversity distances were calculated using the Jensen–Shannon, Bray–Curtis, Generalized UniFrac, and Fast UniFrac algorithms as appropriate [27–30]. With respect to antibody durability and fold-change, taxonomic and functional biomarkers were identified using the linear discriminant analysis (LDA) effect size method [31]. An LDA effect size of ≥ 2.0 was considered significant. Functional profiles were predicted using PICRUSt and MinPath algorithms [32, 33]. Raw reads were quality-checked, and low-quality (< Q25) reads were filtered using Trimmomatic ver. 0.32. After quality control processing, paired-end sequence data were merged using the fastq_mergepairs command in VSEARCH ver. 2.13.4. with the default parameters. Next, the primers were trimmed using the Myers–Miller alignment algorithm at a similarity cut-off of 0.8 [34]. Nonspecific amplicons, such as those not encoding 16S rRNA, were detected using the nhmmer algorithm in the HMMER software package ver. 3.2.1 with hmm profiles [35]. Unique reads were extracted, and redundant reads were clustered with unique reads using the deep-full-length command in VSEARCH [36]. Taxonomic assignment was performed using the EzBioCloud 16S rRNA database with the usearch_global command in VSEARCH, followed by a more precise pairwise alignment [34, 36, 37]. Chimeric reads were filtered to obtain reads with < 97% similarity through reference-based chimeric read detection using the UCHIME algorithm and nonchimeric 16S rRNA database from EzBioCloud [38]. After chimeric filtering, reads not identified to the species level (< 97% similarity) in the EzBioCloud database were compiled, and the cluster_fast command was used for de novo clustering to generate additional OTUs. OTUs with single reads (singletons) were excluded from further analysis [36]. Secondary analyses, including diversity calculations and biomarker discovery, were performed using in-house programs from CJ Bioscience.

### Analysis pipeline for shotgun sequencing

Genomic DNA was extracted from isolates using the FastDNA™ Spin Kit for Soil (MP Biomedicals) according to the manufacturer’s instructions. The concentration of the extracted DNA was quantified using a Qubit 2.0 fluorometer (Invitrogen, Carlsbad, CA, USA). Sequencing libraries were constructed using the NEBNext Ultra II FS DNA Library Prep Kit for Illumina (NEB, Ipswich, MA, USA) according to the manufacturer’s protocol for use with inputs ≤ 100 ng. Metagenome sequencing was performed on the NextSeq 1000 system with 2 × 150 bp paired-end reads using a 300-cycle (NextSeq 1000 Reagent Kit) sequencing kit. The profiling process began by surveying the potential presence of bacterial and archaeal species in each raw metagenomic sample read using Kraken2 [39] and a prebuilt core gene database [40] containing k-mers (k = 35) of reference genomes obtained from the EzBioCloud database [37]. After acquiring a list of candidate species, a custom bowtie2 [41] database was built using the core genes of species identified during the first step. The raw samples were then mapped against the bowtie2 database using the very-sensitive option and a phred33 quality threshold. Samtools [42] was used to convert and sort the output BAM file. The coverage of the mapped reads against the BAM file was obtained using Bedtools [43]. To avoid false positives, an in-house script was used to quantify all reads mapped to a given species only if the total coverage of their core genes was ≥ 25%. Finally, species abundance was calculated using the total number of count reads, while normalized species abundance was calculated using the total length of all core genes.

Taxonomic data were calculated as the species ratio based on the analysis using the Bacteria and Archaea database. In contrast, functional data were obtained as counts per million values based on the analysis using the Kyoto Encyclopedia of Genes and Genomes (KEGG), Enzyme commission, and EggNOG databases [44]. Functional profiling was conducted using HUMAnN (the HMP Unified Metabolic Analysis Network). HUMAnN uses metagenomic data as input and processes them to estimate the functions of the microbial community. This was done by comparing the genetic sequences to a database of known microbial genes and pathways and then inferring the functions of the community [45].

### Statistical analysis

All continuous variables are expressed as the median ± interquartile range (IQR; third IQR–first IQR), whereas categorical variables are presented as numbers (percentages). Nonparametric tests were used to compare the differences between different groups. Statistical analysis was performed using the LDA effect size method to support high-dimensional class comparisons with a particular focus on metagenomic analyses and depicted using the R Statistical Software (version 4.1.2; R Foundation for Statistical Computing, Vienna, Austria) with the ggplot2 and vegan packages.

## Results

**Fig. 1:**
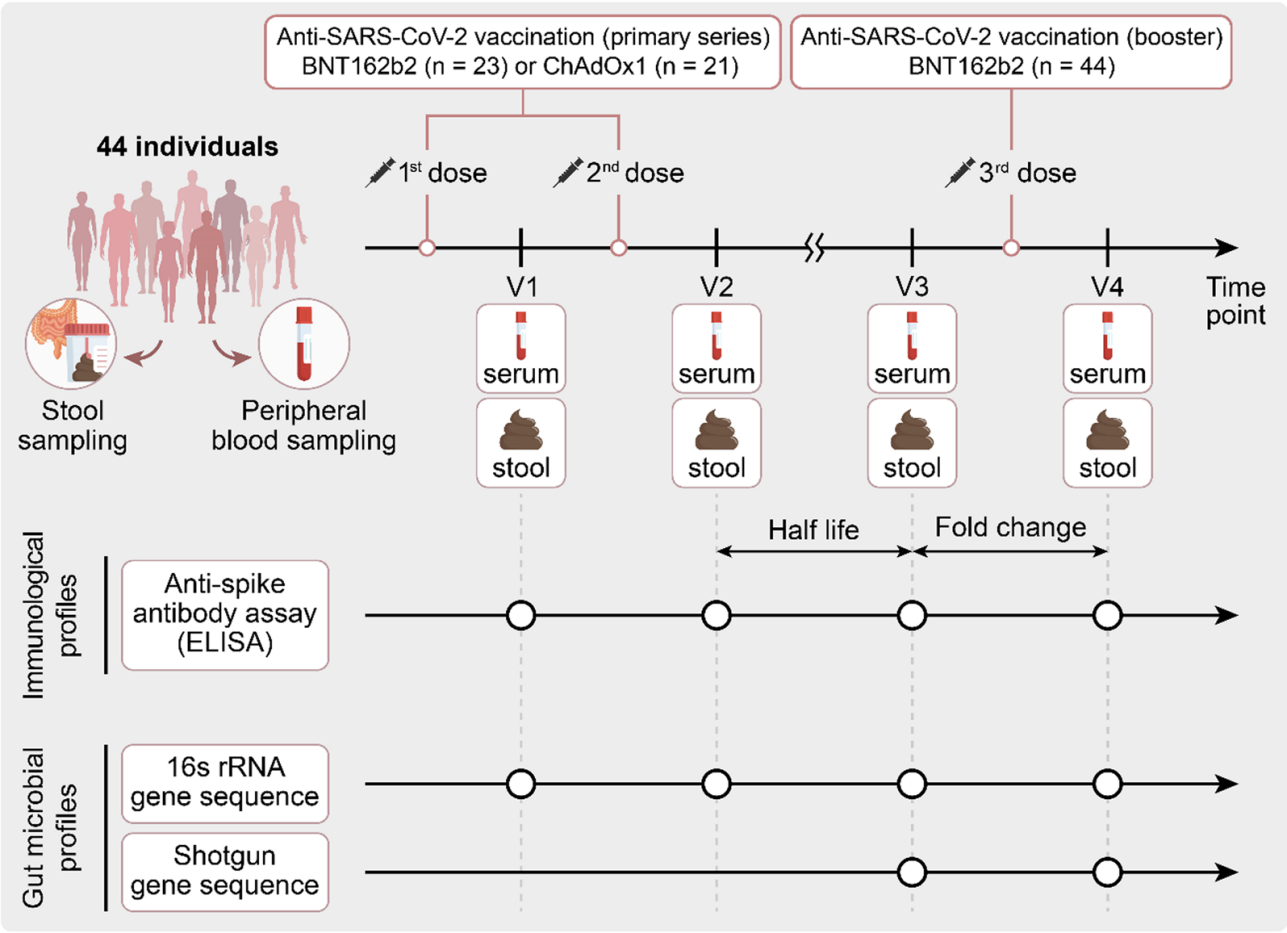
Schematic study diagram

From March to April 2021, we prospectively recruited 44 healthy adults who received the first dose of either the BNT162b2 (*n* = 23) or ChAdOx1 (*n* = 21) vaccine and were scheduled to receive the second dose of the primary vaccination series (Fig. 1). The median age of the study participants was 31 ± 8.5 years, with 75% (33) being women. The baseline geometric mean titer of anti-S IgG of all participants was 98.3 U/mL (Table S1). We analyzed fecal and blood samples at four time points: V1 (prior to the second dose), V2 (3 weeks after the second dose), V3 (6 months after the second dose and prior to the booster dose), and V4 (3 weeks after the BNT162b2 booster dose). After the two-dose primary vaccination series, the median antibody half-life between V2 and V3 was 141.0 d in the BNT162b2 cohort, whereas it was 63.4 d in the ChAdOx1 cohort. Based on the half-life of anti-S IgG antibodies, each vaccination cohort was classified into slow- and fast-decay groups (Tables S2 and S3). After the booster dose vaccination, the BNT162b2 recipients were divided into two groups (high versus low responders) based on the median value (34-fold) of the antibody fold-change from V3 to V4 (Table S4).

Of the 176 stool samples collected, 13 were excluded because of poor quality, leaving 36 (V1), 39 (V2), and 44 (V3 and V4) samples for gut microbiota analysis. Using corresponding blood samples, we evaluated the anti-S humoral immune response. During the follow-up period, the study participants did not show any major changes in their dietary habits (data not shown; a pseudonymized individual study dataset can be made available upon request).

### Association between anti-S antibody half-life and gut microbiota using 16S rRNA sequencing in the BNT162b2 cohort

**Fig. 2:**
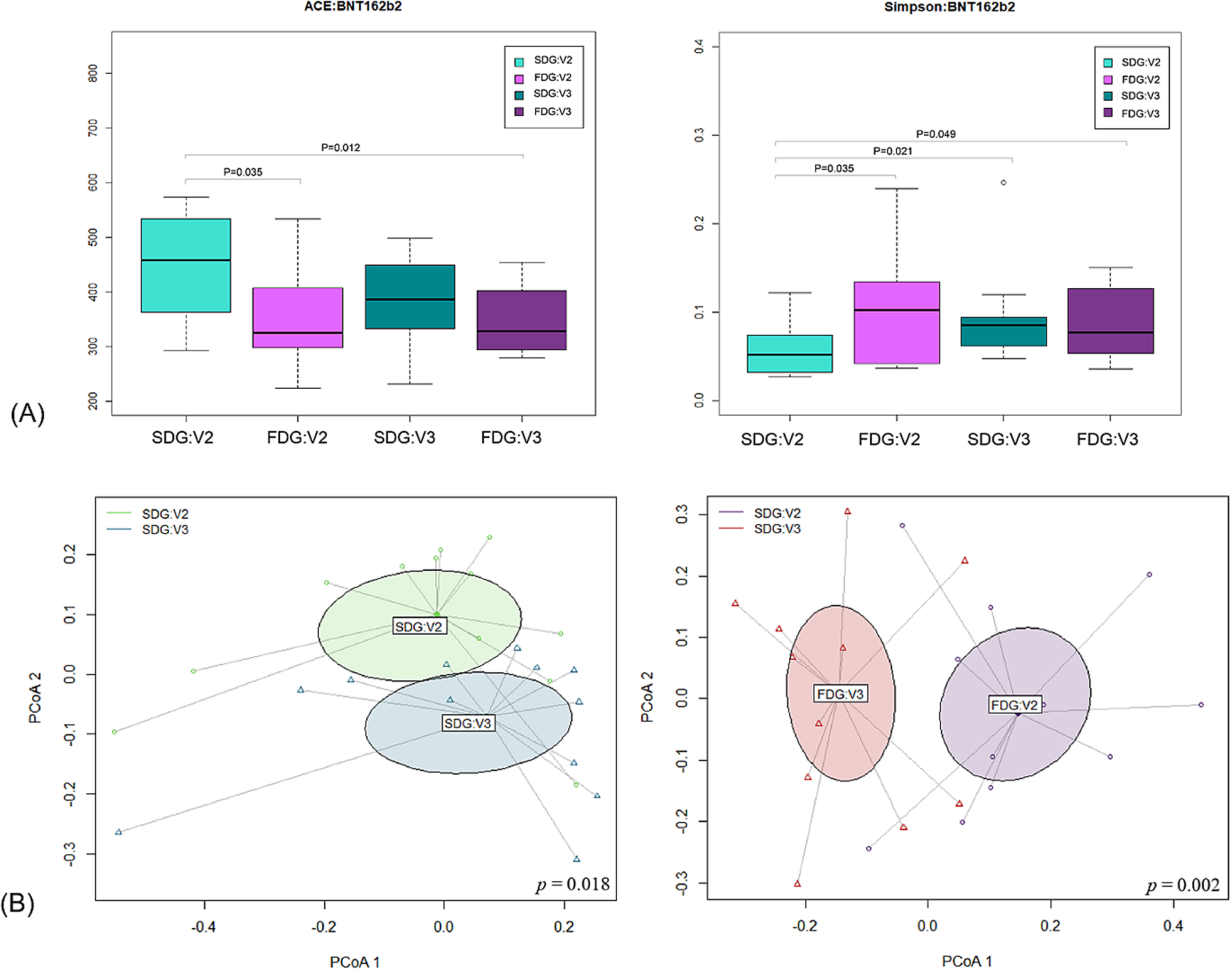
Alpha and beta diversity comparison of the anti-S antibody titer between the slow- and fast-decay groups (BNT162b2 cohort). **A** Alpha diversity (ACE and Simpson). **B** Principal coordinate analysis (PCoA) using the Jensen–Shannon algorithm, excluding unclassified operational taxonomic units (OTUs)

Regarding alpha diversity, we calculated species richness and diversity using the abundance-based coverage estimator (ACE) and Simpson indices, which revealed significant differences between the slow- and fast-decay groups (Fig. 2A). The ACE index in the slow-decay group at V2 was significantly higher than that in the fast-decay group at V2 (*p* = 0.035) and V3 (*p* = 0.012), indicating higher species richness in the former. Similarly, the Simpson index in the slow-decay group at V2 was significantly lower (higher diversity) than that in the fast-decay group at both V2 (*p* = 0.035) and V3 (*p* = 0.049). Interestingly, these differences in the alpha diversity indices between the two groups disappeared six months after the second dose (V3) (ACE index, *p* = 0.176; Simpson index, *p* = 0.712). Analysis using the Jensen–Shannon algorithm, excluding unclassified operational taxonomic units (OTUs) (Fig. 2B), revealed apparent differences in beta diversity values between V2 and V3 in the slow-decay (*p* = 0.018) and fast-decay (*p* = 0.002) groups.

Following the two-dose primary vaccination series, the slow-decay group exhibited distinctly abundant bacterial species in the gut microbiota from stool samples collected 3 weeks after the second dose compared with those in the fast-decay group (Table 1 and Table S5). Certain bacterial species were significantly associated with slower antibody decay in individuals who received the BNT162b2 vaccine. *Faecalibacterium prausnitzii* was the most abundant species, with a linear discriminant analysis (LDA) effect size of 4.26175 (*p* = 0.04095). *Prevotella_uc* and *PAC001304_s*, both from the genus *Prevotella*, showed LDA effect sizes of 4.25488 (*p* = 0.00819) and 4.15907 (*p* = 0.04801), respectively. Another notable species, *Gemmiger formicilis* from the family Ruminococcaceae, had an LDA effect size of 3.91776 (*p* = 0.03896), whereas *Bacteroides dorei*, a member of the family Bacteroidaceae, had an LDA effect size of 3.71884 (*p* = 0.01251). Similarly, *Agathobacter rectalis*, a member of the family Lachnospiraceae, had an LDA effect size of 3.49743 (*p* = 0.04703). In addition, *PAC001173_s*, *PAC000195_s*, *JPJG_s*, *PAC001037_s*, *PAC001187_s*, *PAC001335_s*, and *Bacteroides_uc* showed pronounced LDA effect sizes, indicating their potential contributions to the modulation of vaccine-induced immunity. At the genus level (Table S5), *Prevotella* had an LDA effect size of 4.62838 (*p* = 0.0147), followed by *Faecalibacterium* with an effect size of 4.26036 (*p* = 0.04791). Other significant genera were *Subdoligranulum* (LDA = 3.98239, *p* = 0.01467), *Agathobacter* (LDA = 3.50072, *p* = 0.04703), and *Eubacterium_g23* (LDA = 3.05084, *p* = 0.00962). Finally, the compelling LDA effect sizes of *PAC000195_g*, *PAC000196_g*, *PAC000692_g*, and *PAC001236_g* suggested their potential effect in vaccine-mediated host immune responses.

**Table 1:** Species-level taxonomic markers linked to extended antibody durability following the two-dose primary vaccination series

**Taxon name**	**Taxon rank**	**Taxonomy**	**LDA effect size**	***p-*value**
BNT162b2				
*Faecalibacterium prausnitzii*	Species	Bacteria: Firmicutes: Clostridia: Clostridiales: Ruminococcaceae: *Faecalibacterium*	4.26175	0.04095
*Prevotella_uc*	Species	Bacteria: Bacteroidetes: Bacteroidia: Bacteroidales: Prevotellaceae: *Prevotella*	4.25488	0.00819
*PAC001304_s*	Species	Bacteria: Bacteroidetes: Bacteroidia: Bacteroidales: Prevotellaceae: *Prevotella*	4.15907	0.04801
*Gemmiger formicilis*	Species	Bacteria: Firmicutes: Clostridia: Clostridiales: Ruminococcaceae: *Subdoligranulum*	3.91776	0.03896
*Bacteroides dorei*	Species	Bacteria: Bacteroidetes: Bacteroidia: Bacteroidales: Bacteroidaceae: *Bacteroides*	3.71884	0.01251
*Agathobacter rectalis*	Species	Bacteria: Firmicutes: Clostridia: Clostridiales: Lachnospiraceae: *Agathobacter*	3.49743	0.04703
*PAC001173_s*	Species	Bacteria: Firmicutes: Clostridia: Clostridiales: Ruminococcaceae: *Subdoligranulum*	3.14941	0.04366
*PAC000195_s*	Species	Bacteria: Firmicutes: Clostridia: Clostridiales: Lachnospiraceae: *PAC000195_g*	3.1406	0.0476
*JPJG_s*	Species	Bacteria: Firmicutes: Clostridia: Clostridiales: Ruminococcaceae: *Oscillibacter*	3.01913	0.04765
*PAC001037_s*	Species	Bacteria: Firmicutes: Clostridia: Clostridiales: Ruminococcaceae: *Oscillibacter*	2.6809	0.01235
*PAC001187_s*	Species	Bacteria: Firmicutes: Clostridia: Clostridiales: Ruminococcaceae: *Oscillibacter*	2.6189	0.00492
*PAC001335_s*	Species	Bacteria: Firmicutes: Clostridia: Clostridiales: Lachnospiraceae: *PAC000196_g*	2.55763	0.03297
*Bacteroides_uc*	Species	Bacteria: Bacteroidetes: Bacteroidia: Bacteroidales: Bacteroidaceae: *Bacteroides*	2.53203	0.03742
*Bacteroides xylanisolvens*	Species	Bacteria: Bacteroidetes: Bacteroidia: Bacteroidales: Bacteroidaceae: *Bacteroides*	2.38654	0.04201
*PAC001229_s*	Species	Bacteria: Firmicutes: Clostridia: Clostridiales: Lachnospiraceae: *PAC000692_g*	2.32714	0.02256
*PAC001162_s*	Species	Bacteria: Firmicutes: Clostridia: Clostridiales: Ruminococcaceae: *Sporobacter*	2.23478	0.04096
*PAC001306_s*	Species	Bacteria: Firmicutes: Clostridia: Clostridiales: Ruminococcaceae: *Sporobacter*	2.21794	0.04844
*PAC001467_s*	Species	Bacteria: Firmicutes: Clostridia: Clostridiales: Lachnospiraceae: *PAC000692_g*	2.13406	0.01007
*PAC001130_s*	Species	Bacteria: Firmicutes: Clostridia: Clostridiales: Ruminococcaceae: *Oscillibacter*	2.12692	0.03535
*PAC001236_s*	Species	Bacteria: Firmicutes: Clostridia: Clostridiales: Mogibacterium_f: *PAC001236_g*	2.11986	0.01768
*PAC001449_s*	Species	Bacteria: Firmicutes: Clostridia: Clostridiales: Lachnospiraceae: *PAC001043_g*	2.08894	0.01923
ChAdOx1				
*Escherichia coli*	Species	Bacteria: Proteobacteria: Gammaproteobacteria: Enterobacterales: Enterobacteriaceae: *Escherichia*	3.85796	0.00937

LDA, linear discriminant analysis. LDA effect size and *p*-value are expressed as values of V2

Following the BNT162b2 administration, we performed 16S rRNA gene sequencing analysis of the gut microbiota and identified specific functional marker proteins that may affect antibody decay rates (Table S6). Interestingly, we found that the key functional markers were starch-binding outer membrane proteins from the SusD/RagB family (LDA = 3.02715, *p* = 0.02956), RNA polymerase sigma-70 factor from the ECF subfamily (LDA = 2.89815, *p* = 0.03486), and iron complex outer membrane receptor protein (LDA = 2.89246, *p* = 0.01222). Other noteworthy markers included the TonB-dependent starch-binding outer membrane protein SusC (LDA = 2.83329, *p* = 0.03486), an uncharacterized protein (LDA = 2.58362, *p* = 0.00686), a multidrug resistance protein of the MATE family (LDA = 2.56360, *p* = 0.04095), a HlyD family secretion protein (LDA = 2.53494, *p* = 0.01470), and integrase/recombinase XerD (LDA = 2.51074, *p* = 0.04791).

Further investigation into the functional capabilities of the microbiota revealed specific modules and pathways that may influence vaccine immunogenicity. PICRUSt analysis revealed that F-type ATPase (LDA = 2.93490, *p* = 0.00686) and an incomplete reductive citrate cycle (LDA = 2.69979, *p* = 0.04095) were significantly associated with a longer antibody half-life. MinPath analysis highlighted the positive relationship between a longer half-life and lysine biosynthesis via the aromatic amino acid pathway (LDA = 2.74262, *p* = 0.01470) and formaldehyde assimilation via the serine pathway (LDA = 2.73185, *p* = 0.01761). Furthermore, PICRUSt analysis identified the lysosome (LDA = 2.53934, *p* = 0.02956) and photosynthesis (LDA = 2.46671, *p* = 0.00835) pathways, whereas MinPath analysis revealed the vitamin B6 metabolism (LDA = 3.15938, *p* = 0.01382) and lipopolysaccharide biosynthesis (LDA = 3.09433, *p* = 0.01162) pathways to be more abundant in the slow-decay group.

### Association between anti-S antibody half-life and gut microbiota using 16S rRNA sequencing in the ChAdOx1 cohort

Unlike the BNT162b2 cohort, we did not detect any significant differences in alpha and beta diversity between groups in the ChAdOx1 cohort (data not shown). Postvaccination analysis of stool samples collected from ChAdOx1 recipients 3 weeks after the second dose revealed the presence of distinct gut bacterial species associated with longer antibody half-lives (Table 1 and Table S5). Specifically, *Escherichia coli* was associated with slow antibody decay (LDA effect size of 3.85796, *p* = 0.00937). At the genus level, *Alistipes* was significantly more abundant, with an LDA of 4.46179 (*p* = 0.03844), followed by *Escherichia* (LDA = 3.8579, *p* = 0.00937), *Parabacteroides* (LDA = 3.85644, *p* = 0.01235), and *Enterococcus* (LDA = 3.23579, *p* = 0.04323).

Detailed 16S rRNA gene analysis of samples from ChAdOx1 recipients revealed specific functional markers based on their gut microbiota (Table S6). In particular, we identified the insertion element IS1 protein InsB (LDA = 2.47573, *p* = 0.00937) and transposase (LDA = 2.44395, *p* = 0.00937) as primary ortholog markers reflecting a longer antibody half-life. Further exploration of specific modules and pathways showed that ADP-L-glycero-D-mannohypertose biosynthesis (LDA = 2.50396, *p* = 0.01614) and photorespiration (LDA = 2.40116, *p* = 0.04331) were correlated with a longer antibody half-life in PICRUSt analysis, whereas the trehalose biosynthetic pathway (LDA = 2.70821, *p* = 0.00937) was identified in MinPath analysis.

### Association between gut microbiota and acute immune response after BNT162b2 booster vaccination—comparison of the fold changes of anti-S antibody titers

To evaluate acute antibody responses after the BNT162b2 booster vaccination, we divided vaccine recipients into two groups (high versus low responders) based on the median value (34-fold) of the antibody fold-change.

Using 16S rRNA gene sequencing, we did not detect any significant differences in alpha and beta diversity between the two groups (data not shown). Table 2 shows the gut microbiota that potentially contributed to significant fold changes in antibody titers following booster vaccination. In species-level analysis, *F. prausnitzii*, a member of the family Ruminococcaceae, was abundant in high responders, exhibiting the highest LDA effect size of 4.40539 (*p* = 0.03998). *Peptoniphilus duerdenii* (LDA effect size 2.98548, *p* = 0.03822) of the family Peptoniphilaceae, JRNA_s (LDA effect size 2.30296, *p* = 0.04727) of the family Mogibacterium_f, PAC001048_s (LDA effect size 2.18111, *p* = 0.02423) of the family Ruminococcaceae, and *Anaerococcus provencensis* (LDA effect size 2.16254, *p* = 0.01913) of the family Peptoniphilaceae were also significantly enriched in high responders. At the genus level (Table S7), *Faecalibacterium*, belonging to the family Ruminococcaceae of the order Clostridiales in the phylum Firmicutes, was abundant in high responders, exhibiting the highest LDA effect size of 4.40882 (*p* = 0.03998). Similarly, *Clostridium* (LDA effect size 3.65726, *p* = 0.02734) and *Agathobaculum* (LDA effect size 2.41073, *p* = 0.03071), both of the order Clostridiales, were associated with a higher antibody fold-change.

**Table 2:** Species-level taxonomic markers associated with high fold increase in antibody titer following BNT162b2 booster vaccination

Taxon name	Taxon rank	Taxonomy	LDA effect size	*p-*value
*Faecalibacterium prausnitzii*	Species	Bacteria: Firmicutes: Clostridia: Clostridiales: Ruminococcaceae: *Faecalibacterium*	4.40539	0.03998
*Peptoniphilus duerdenii*	Species	Bacteria: Firmicutes: Tissierellia: Tissierellales: Peptoniphilaceae: *Peptoniphilus*	2.98548	0.03822
*JRNA_s*	Species	Bacteria: Firmicutes: Clostridia: Clostridiales: Mogibacterium_f: *JRNA_g*	2.30296	0.04727
*PAC001048_s*	Species	Bacteria: Firmicutes: Clostridia: Clostridiales: Ruminococcaceae: *PAC000672_g*	2.18111	0.02423
*Anaerococcus provencensis*	Species	Bacteria: Firmicutes: Tissierellia: Tissierellales: Peptoniphilaceae: *Anaerococcus*	2.16254	0.01913

LDA, linear discriminant analysis. LDA effect size and *p*-value are expressed as values of V3

**Fig. 3:**
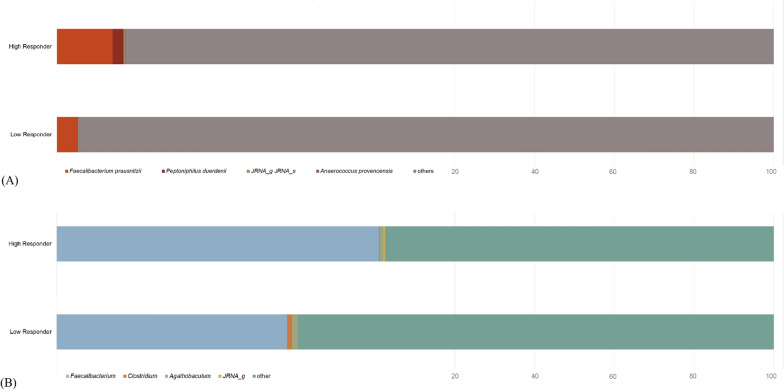
Shotgun sequencing-based compositional differences in gut microbiota between high and low responders after the BNT162b2 booster vaccination. **A** Species level. **B** Genus level

Shotgun sequencing-based microbiota analysis yielded consistent results (Fig. 3). In particular, the species *F. prausnitzii* (high: 2.8%, low: 1.05%) and *P. duerdenii* (high: 0.55%, low: 0%) and the genus *Faecalibacterium* (high: 15.7%, low: 11.2%) were more abundant in high than in low responders.

### Correlation between abundance of specific gut microbiota and antibody half-life or titer fold changes

To elucidate the correlations between species-level taxonomic markers (with an LDA effect size > 2.0) and antibody half-life in the BNT162b2 and ChAdOx1 vaccine cohorts, we conducted Spearman’s correlation analysis. Significance was determined using criteria of absolute correlation coefficients > 0.4 and p-values < 0.05 (Figure S1). In the BNT162b2 cohort, we observed notable positive correlations between the antibody half-life and *F. prausnitzii*, *Prevotella_uc*, PAC001304_s, *A. rectalis*, PAC001173_s, JPJG_s, PAC001187_s, and PAC001335_s. In contrast, in the ChAdOx1 cohort the antibody half-life was only positively correlated with *E. coli*. Additionally, *F. prausnitzii* and JRNA_s were positively correlated with the fold changes in antibody titers.

### Shotgun sequencing-based functional gene analysis—comparison of the fold changes of anti-S antibody titers following BNT162b2 booster vaccination

A detailed shotgun-based analysis of BNT162b2 booster vaccine recipients revealed specific functional markers in the gut microbiome (Table 3). In particular, the translation initiation factor IF-1 (LDA = 2.5893, *p* = 0.0433), ATP-dependent Clp protease, protease subunit (LDA = 2.5134, *p* = 0.0341), large subunit ribosomal protein L35 (LDA = 2.4083, *p* = 0.2066), ribosome-binding factor A (LDA = 2.3489, *p* = 0.0341), bacterial/archaeal transporter family-2 protein (LDA = 2.21821, *p* = 0.0341), beta-lactamase class A TEM [EC:3.5.2.6] (LDA = 2.2132, *p* = 0.0178), and excinuclease ABC subunit B (LDA = 2.187, *p* = 0.03433) were more abundant in high than in low responders. Except for one marker (bacterial/archaeal transporter family-2 protein), high responders showed higher abundances of these primary ortholog markers. Using the Clusters of Orthologous Genes (COG) database, we identified the IstB domain ATP-binding protein (LDA = 2.6756, *p* = 0.0421) as an important marker of an increased antibody response. This gene is associated with DNA replication, membrane fusion, and gene expression regulation [46]. Furthermore, EC:2.1.1.37 (cytosine-specific DNA methyltransferase) and EC:4.2.3.4 (3-dehydroquinate synthase) were enriched in high responders compared with those in low responders.

**Table 3:** Functional markers associated with high fold increase in antibody titer following BNT162b2 booster vaccination

	Meta shotgun				
	Definition	High V4	Low V4	LDA effect size	*p-*value
COG (eggNoG)					
COG1484	IstB domain protein ATP-binding protein	0.116	0.0421	2.6756	0.0421
Ortholog					
K02518	Translation initiation factor IF-1	0.2564	0.1872	2.5893	0.0433
K01358	ATP-dependent Clp protease, protease subunit [EC:3.4.21.92]	0.168	0.1052	2.5134	0.0341
K02916	Large subunit ribosomal protein L35	0.2111	0.1582	2.4083	0.0266
K02834	Ribosome-binding factor A	0.133	0.0893	2.3489	0.0341
K09936	Bacterial/archaeal transporter family-2 protein	0.0161	0.0415	2.2182	0.0341
K18698	Beta-lactamase class A TEM [EC:3.5.2.6]	0.0412	0	2.2132	0.0178
K03702	Excinuclease ABC subunit B	0.1087	0.0772	2.187	0.0433
EC (enzyme commission)					
2.1.1.37	DNA (cytosine-5-)-methyltransferase	0.1886	0.1208	2.4818	0.1208
4.2.3.4	3-dehydroquinate synthase	0.1684	0.1199	2.3975	0.1199

## Discussion

In this longitudinal cohort study of SARS-CoV-2 vaccine recipients, the longevity of anti-S antibodies was significantly associated with the gut microbiota composition. Specifically, *F. prausnitzii*, *Prevotella_uc*, and PAC001304_s from the genera *Prevotella*, as well as *G. formicilis*, *B. dorei*, and *A. rectalis* were associated with a significantly prolonged antibody half-life after SARS-CoV-2 mRNA vaccination. Analysis of the associations between gut microbiota species and fold changes in antibody titers after BNT162b2 booster vaccination revealed that, in comparison to low responders, high responders had a significantly higher abundance of *F. prausnitzii*, which activates the metabolic pathways EC:2.1.1.37 (cytosine-specific DNA methyltransferase) and EC:4.2.3.4 (3-dehydroquinate synthase).

Antibody longevity is influenced by long-lived plasma cells, which are mainly located in the bone marrow, while the magnitude of the acute antibody response to booster vaccinations is likely determined by memory B-cells. Both long-lived plasma and memory B-cells are influenced by the interaction of B-cells, follicular helper T (Tfh) cells, and follicular dendritic cells in the germinal center of B-cell follicles. Kim et al. discovered that short-chain fatty acids (SCFAs) facilitate Tfh cell differentiation both in vitro and in vivo by upregulating cellular metabolism in activated T-cells [47]. Moreover, SCFAs, notably butyrate, have been shown to increase the population of Foxp3 + regulatory T-cells (Tregs) in the colon [48, 49]. Interestingly, under specific conditions, certain Tregs can be converted into Tfh cells within Peyer’s patches [50, 51].

SCFAs promote antibody production via various mechanisms that profoundly affect the human immune system. First, SCFAs effectively enhance the cellular metabolism of B-cells, providing the necessary energy and building blocks for the activation, differentiation, and antibody production of B-cells. In addition, SCFAs increase mitochondrial energy production and glycolysis, which are essential for plasma cell differentiation [52]. Second, SCFAs are involved in the regulation of cellular metabolic pathways as they are converted to acetyl-CoA, which is used for energy production and fatty acid synthesis. In SCFA-treated B-cells, the levels of acetyl-CoA and lipid droplets increase, thereby fostering B-cell differentiation and antibody production [47]. Third, SCFAs regulate the expression of key genes involved in B-cell differentiation. In particular, they upregulate the expression of the immunoglobulin gene family members and that of genes such as Xbp1, Irf4, and Aicda, which are vital for plasma B-cell differentiation and Ig class switch recombination [47]. Finally, SCFAs activate T-cells and phagocytes, enhancing antibody responses [49, 53–55]. Specifically, SCFAs promote the generation and function of Tfh cells, which play a key role in B-cell differentiation and the germinal center reaction, leading to the production of high-affinity antibodies and long-term humoral responses [47]. In this study, the group with a longer antibody half-life (slow decay) within the BNT162b2 cohort was significantly enriched in *F. prausnitzii*, which is one of the most important butyrate-producing species [56]. Other SCFA-producing bacterial agents, including members of the genera *Prevotella* and *Bacteroides,* as well as the families Ruminococcaceae and Lachnospiraceae, were also significantly abundant in the slow decay group.

Moreover, *F. prausnitzii* is one of the most abundant bacteria present in the healthy human gut [57], where it plays a crucial role in the induction of colonic Treg cells [58]. Tregs modulate the immune response and maintain immune homeostasis [59]. Therefore, in individuals with a high abundance of *F. prausnitzii*, the unnecessary nonspecific activation of T-cells might be suppressed, and chronic low-grade inflammation might be reduced, ensuring an appropriate immune response upon exposure to antigens. These findings align with the concept of inflammaging (age-related chronic inflammation), underscoring the decline in the numbers of anti-inflammatory bacteria such as *F. prausnitzii* in older individuals [60].

Our study suggested a potential association between a high abundance of *E. coli* in the gut and an extended antibody half-life in ChAdOx1 vaccine recipients. Antibody half-life is modulated by several factors, including the magnitude of the immune response, antibody production efficiency, and degradation kinetics. *E. coli* secretes signaling molecules with notable immunostimulatory properties such as LTA1 (A1 domain of heat-labile enterotoxin), monophosphoryl lipid A, and maltose-binding protein, which are instrumental in dendritic cell (DC) activation [61–63]. Upon activation, DCs relocate to the proximate lymph nodes to present the antigens to T-cells, potentially initiating T- and B-cell activation. Furthermore, *E. coli* has been suggested to amplify the activity of proinflammatory CD4 + T-cells, potentially heightening the responsiveness to vaccine antigens [64]. In the case of adenovirus-vector vaccines, the quantitative and qualitative activity of DCs may be particularly important in inducing a strong and durable immune response [65].

In the slow-decay group of the BNT162b2 cohort, a notable activation of Kyoto Encyclopedia of Genes and Genomes (KEGG) orthologs K21572 and K21573, which are starch-binding outer membrane proteins, was observed. Specifically, K21572 was associated with the SusD/RagB family, whereas K21573 was associated with SusC. These proteins play pivotal roles in starch binding and transport. A significant proportion of Bacteroidetes strains, particularly those belonging to the genera *Prevotella* and *Bacteroides*, have evolved to specialize in the breakdown of complex carbohydrates [66, 67]. The observed increase in the abundance of these microbial species in our taxonomic analysis may be correlated with the heightened activation of the above-mentioned KEGG orthologs. Furthermore, the metabolism of starch and other intricate carbohydrates by gut microbes often leads to SCFA production [68]. Therefore, K21572 and K21573 may influence SCFA synthesis, indirectly affecting antibody longevity.

Memory T-cells, particularly memory Tfh cells, assist in activating B-cells, which then differentiate into antibody-producing plasma cells [69]. Therefore, a strong memory T-cell response can support rapid and robust antibody production during subsequent encounters with a pathogen. In 16S gene analysis of the BNT162b2 cohort, we observed that the ortholog K01897, together with module M00157 and pathway ko00540, which are functional markers of oxidative phosphorylation and lipid metabolism, respectively, were notably activated in the slow antibody decay group. Memory T-cells typically exhibit a metabolic profile that relies more on oxidative phosphorylation and lipid metabolism, whereas activated effector T-cells are more glycolysis-dependent [70]. Given this distinction, our findings suggested that the activation of K01897, M00157, and ko00540 in the slow-decay group may be associated with the activation of memory T-cells.

In shotgun sequencing-based functional gene analysis, we found that the aromatic amino acid synthesis pathways serve as a link between the gut microbiota and the vaccine immune response. The synthesis of aromatic amino acids (L-tyrosine, L-phenylalanine, and L-tryptophan), which are essential for protein biosynthesis in all living organisms, is accomplished via the shikimate and chorismate pathways. These metabolic pathways exist in some protists, bacteria, fungi, and plants, but not in animals [71]. In particular, the shikimate pathway connects primary metabolism and aromatic amino acid biosynthesis. The first enzyme in the shikimate pathway, 3-deoxy-D-arabino-heptulosonate 7-phosphate synthase (DAHPS), generates 3-deoxy-D-arabino-heptulosonate 7-phosphate (DAHP), while the second enzyme, 3-dehydroquinate synthase (DHQS), converts DAHP into 3-dehydroquinate. DAHPSs are divided into two types according to their amino acid homology: type I DAHPS derived from microorganisms, and type II DAHPS found in plants and some microorganisms [72, 73]. *F. prausnitzii* activates the shikimate and phenylpropanoid pathways by inducing the activity of 3-dehydroquinate synthase (EC:4.2.3.4), which is involved in the early increase in the expression of *DAHPS* and phenylalanine ammonia lyase (*PAL*). In this study, shotgun-based analysis revealed that *F. prausnitzii* and EC:4.2.3.4 were more abundant in high responders to the BNT162b2 booster vaccination. In plants, the expression of *DAHPS1* is strongly induced within 1 h of wound infection, whereas the expression of *PAL* has been reported to play a major role in inducing resistance to cassava brown streak disease by pathogenic viruses, reflecting early immune responses [74, 75]. In contrast, EC:2.1.1.37 (cytosine-specific DNA methyltransferase), another enriched enzyme in high responders, is known to be involved in gene expression, genome protection against selfish DNA developmental regulation, and T-cell development [76]. Furthermore, six ortholog markers, which were more abundant in high responders, are also involved in maintaining and regulating cellular functions, such as protein synthesis, DNA damage removal, and resynthesis. Considering the results of taxonomic and functional analyses, including enzyme and ortholog analyses (Table 3), the gut microbiome may be significantly associated with the vaccine immune response at the cellular level.

As discussed above so far, the gut microbiota composition might play an important role in modulating the process of immune response after vaccination. Growing evidences have shown that the abundance of phylum Actinobacteria is consistently associated with good vaccine immune responses [77]. Some genera and species of Firmicutes and Proteobacteria is also known to affect vaccine immune responses [77]. Conversely, the composition of gut microbiome is also affected by vaccination as shown among the BNT162b2 recipients in this study. Vaccination might induce changes in the gut microbiota composition, thereby influencing vaccine immunogenicity. A recent study reported that both inactivated and mRNA SARS-CoV-2 vaccination led to lower gut microbial diversity with the relatively high abundance of *Bacteroides caccae* and low abundance of *Coprococcus comes, Dorea longicatena and Ruminococcus obeum* [18]. Several studies have highlighted the bidirectional relationship between the gut microbiota and vaccine efficacy, emphasizing that the gut microbiota can influence the immune response to vaccines—including cholera, rotavirus, hepatitis B, tetanus, and influenza vaccines, not limited to the COVID-19 vaccine—and that vaccination itself can also lead to changes in the gut microbiota [78–80]. It is uncertain whether the composition of gut microbiota is influenced mainly by the vaccine platform or by the antigen itself in relation to the immune response pattern.

This study had several strengths, including both short- and long-term assessments of vaccine immunogenicity and its association with the gut microbiome. Moreover, the linkage between the microbiota and functional pathways could be confirmed more clearly by performing both 16S rRNA and shotgun sequencing. However, this study also had some limitations. First, as this was an observational cohort study, establishing causality between specific microbiota/metabolites and vaccine immune responses was not feasible. Second, the participants in this study were healthy individuals; we did not include groups of older individuals or those with chronic medical conditions. Finally, the sample size was limited due to the rapidly evolving pandemic situation. It was necessary to exclude individuals who had contracted COVID-19 and to enroll participants who received the COVID-19 vaccine within a short timeframe that aligned with the study schedule. These limitations should be addressed in future studies to enable the investigation of causality in a more diverse cohort of participants.

In conclusion, the longevity of COVID-19 vaccine immunity was significantly associated with the composition of the gut microbiota. We showed for the first time the beneficial impact of *F. prausnitzii* in short and long-term vaccine immune responses and identified several intermediate metabolites and enzymes as potential postbiotic candidates for vaccine microbiome adjuvants. The results of this study provide evidence for a gut microbiome-based personalized approach for enhancing vaccine efficacy.

## Supplementary Information

Additional file 1.

## Data Availability

The study data are not publicly available owing to ethical and regulatory restrictions on participant privacy. However, a pseudonymized individual study dataset will be made available from the corresponding author upon request.

## Abbreviations

ACE: Abundance-based coverage estimator
COG: Clusters of Orthologous Genes
HUMAnN: HMP Unified Metabolic Analysis Network
KEGG: Kyoto Encyclopedia of Genes and Genomes
LDA: Linear discriminant analysis
MTP: Microbiome Taxonomic Profiling
NRF: National Research Foundation
OTU: Operational taxonomic units
PAL: Phenylalanine ammonia lyase
PCoA: Principal coordinate analysis
SCFA: Short-chain fatty acids
