# Supplementary material for: The gut microbiota modifies antibody durability and booster responses after SARS-CoV-2 vaccination
Source: J Transl Med. 2024 Sep 6;22:827. doi: 10.1186/s12967-024-05637-2 (PMC11380214; doi:10.1186/s12967-024-05637-2)
Supplement: Supplementary file 1 — Additional file 1. [file 12967_2024_5637_MOESM1_ESM.docx]

Supporting information

**The Gut Microbiota Modifies Antibody Durability and Booster Responses after SARS-CoV-2 Vaccination**

*Hye Seong, Jin Gu Yoon, Eliel Nham, Yu Jung Choi, Ji Yun Noh, Hee Jin Cheong, Woo Joo Kim, Eui Ho Kim, Chulwoo Kim, Young-Hee Han, Sooyeon Lim*, and Joon Young Song**

**This file includes:**

Supplementary Methods

Supplementary Fig. 1

Supplementary Tables 1 to 7

Supplementary References

**Supplementary Methods**

**Microbiological analysis of 16S rRNA**

For amplification of bacterial 16S rRNA, the following fusion primers were used: 341F (5ʹ-AATGATACGGCGACCACCGAGATCTACAC-XXXXXXXXTCGTCGGCAGCGTC-AGATGTGTATAAGAGACAG-CCTACGGGNGGCWGCAG-3ʹ; the underlined sequence indicates the target region primer) and 805R (5ʹ-CAAGCAGAAGACGGCATACGAGAT-XXXXXXXXGTCTCGTGGGCTCGG-AGATGTGTATAAGAGACAG-GACTACHVGGGTATCTAATCC-3ʹ). Fusion primers were constructed in the following order: P5 (P7) graft binding, i5 (i7) index, Nextera consensus, sequencing adaptor, and target region sequence. PCR amplification was carried out under the following conditions: initial denaturation at 95 °C for 3 min followed by 25 cycles of denaturation at 95 °C for 30 s, primer annealing at 55 °C for 30 s, and extension at 72 °C for 30 s, with a final elongation step at 72 °C for 5 min.

**Analysis for shotgun sequencing**

Beta diversity provides an understanding of the differences in the microbial community between samples. The Jensen-Shannon distance is used for statistical analysis, while an ordination analysis such as Principal Coordinates ordination analysis (PCoA) is used for visualization [1]. The HMP Unified Metabolic Analysis Network (HUMAnN) pipeline method is used for efficiently and accurately profiling the abundance of microbial metabolic pathways and other molecular functions from metagenomic or metatranscriptomic sequencing data [2].

**Supplementary Figures**

**Figure S1. Spearman’s correlation of associated microbiota with antibody half-life and titer fold changes.** A correlation analysis was carried out to assess the relationship between species-level taxonomic markers and antibody half-life in the BNT162b2 (A) and ChAdOx1 cohorts (B). Associations between the composition of enriched microbiota species and fold changes in antibody titers were examined (C). Significance was determined by absolute correlation coefficients exceeding 0.4 and p-values < 0.05. The strength of correlation is represented by a color gradient, with blue indicating negative correlation and red indicating positive correlation. *p < 0.05; **p = 0.01–0.001; ***p < 0.001.

**(A)**


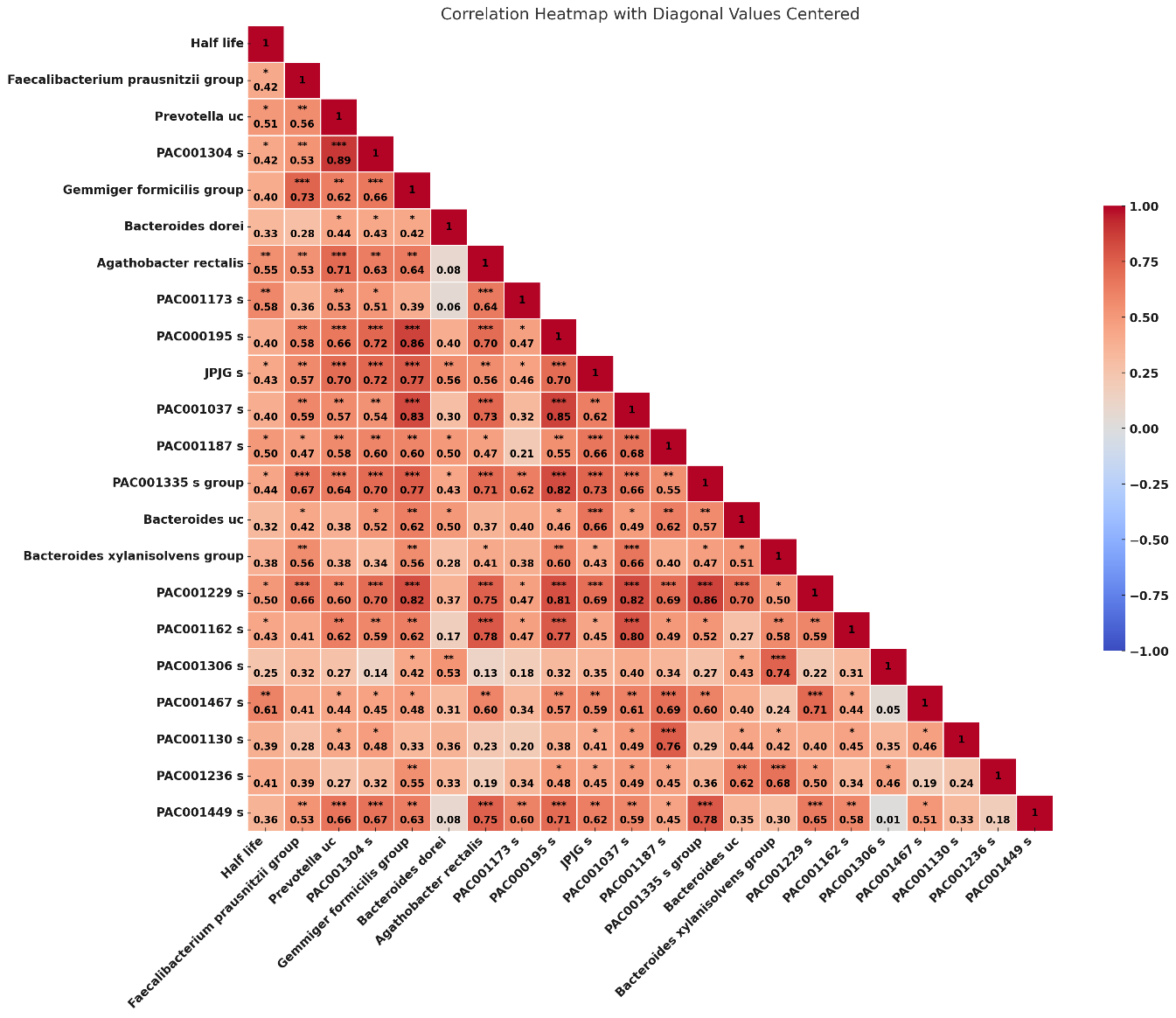


**(B)**


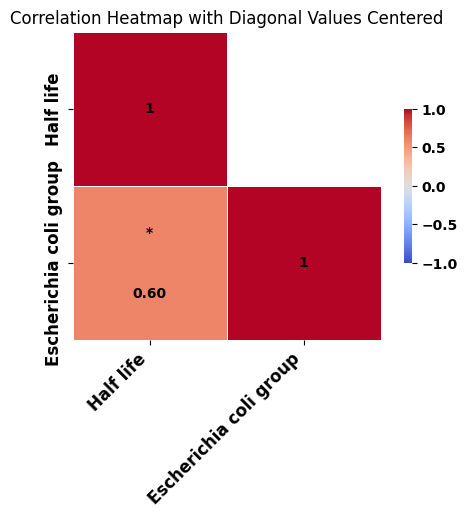


**(C)**


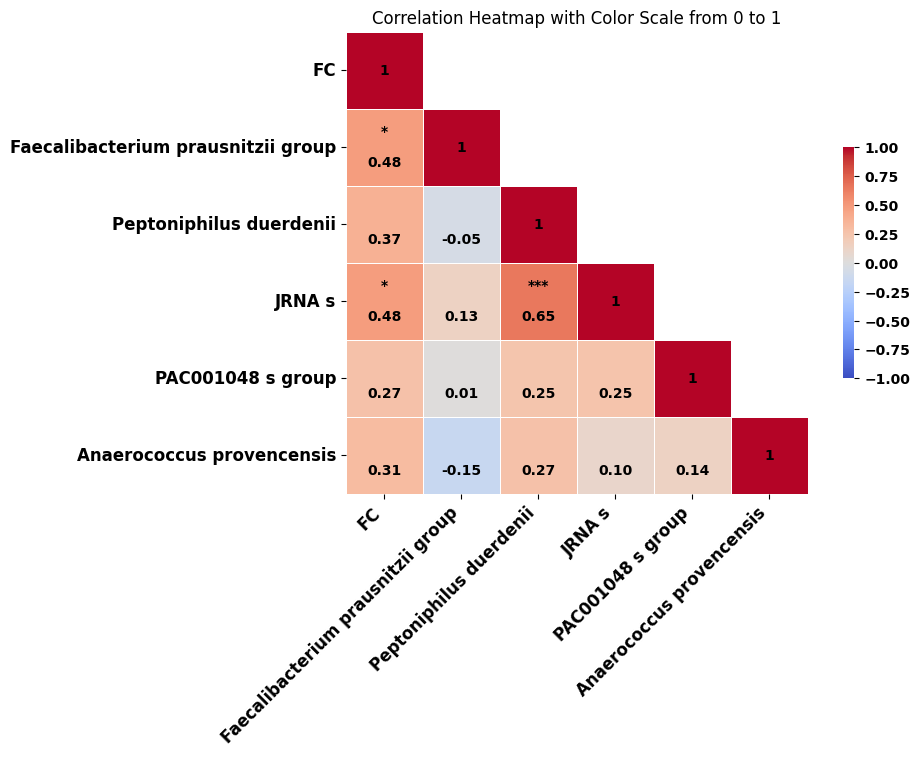


**Supplementary Tables**

| **Table S1. Demographic and laboratory profiles of study participants** | | | | |
| --- | --- | --- | --- | --- |
| **Characteristics** | **V1**  (*n* = 44) | **V2**  (*n* = 44) | **V3**  (*n* = 44) | **V4**  (*n* = 44) |
| **Age (years)** | 36.4 ± 10.2 |  |  |  |
| **Sex (women) (%)** | 33 (75.0) |  |  |  |
| **BMI (kg/m^2^)** | 22.7 ± 3.8 |  |  |  |
| **Anti-SARS-CoV-2 S IgG (U/mL)** | 98.3 ± 113.1 | 2364.0 ± 1918.1 | 713.3 ± 534.8 | 23453.2 ± 13941.5 |
| **Laboratory test results** |  |  |  |  |
| WBC (10^3^/μL) | 6.1 ± 1.4 | 5.8 ± 1.4 | 6.0 ± 1.6 | 5.7 ± 1.3 |
| ANC (/μL) | 3212.8 ± 1048.1 | 3110.6 ± 1032.5 | 3276.6 ± 1297.7 | 3081.8 ± 1127.8 |
| Hemoglobin (g/dL) | 13.5 ± 1.0 | 13.4 ± 1.1 | 13.6 ± 1.2 | 13.5 ± 1.3 |
| MCV (fL) | 91.0 ± 4.5 | 91.0 ± 4.6 | 90.5 ± 4.4 | 90.2 ± 4.4 |
| MCH (pg) | 30.3 ± 1.8 | 30.3 ± 1.9 | 29.8 ± 4.5 | 30.3 ± 1.8 |
| MCHC (g/dL) | 33.3 ± 0.6 | 33.3 ± 0.7 | 33.6 ± 0.6 | 33.6 ± 0.7 |
| Platelet count (10^3^/μL) | 272.6 ± 64.6 | 268.6 ± 61.8 | 267.5 ± 61.2 | 264.4 ± 61.0 |
| BUN (mg/dL) | 12.2 ± 3.6 | 12.0 ± 2.8 | 12.1 ± 2.8 | 12.0 ± 3.7 |
| Creatinine (mg/dL) | 0.7 ± 0.1 | 0.7 ± 0.1 | 0.7 ± 0.1 | 0.7 ± 0.1 |
| Albumin (g/dL) | 4.6 ± 0.2 | 4.5 ± 0.2 | 4.5 ± 0.2 | 4.5 ± 0.2 |
| HDL cholesterol (mg/dL) | 64.3 ± 14.8 | 65.1 ± 15.6 | 66.0 ± 15.2 | 66.0 ± 15.5 |
| LDL cholesterol (mg/dL) | 111.2 ± 26.3 | 107.8 ± 25.9 | 107.1 ± 27.4 | 105.5 ± 28.0 |
| Triglyceride (mg/dL) | 104.2 ± 58.2 | 109.9 ± 80.4 | 94.0 ± 49.8 | 124.2 ± 158.8 |
| AST (IU/L) | 24.2 ± 8.0 | 25.0 ± 8.6 | 25.5 ± 15.6 | 30.8 ± 36.9 |
| ALT (IU/L) | 24.1 ± 27.1 | 24.2 ± 21.2 | 24.5 ± 23.9 | 39.9 ± 100.3 |
| GGT (IU/L) | 21.8 ± 26.8 | 20.0 ± 14.4 | 20.8 ± 20.3 | 21.8 ± 19.4 |
| Total bilirubin (mg/dL) | 0.7 ± 0.3 | 0.7 ± 0.3 | 0.6 ± 0.3 | 0.6 ± 0.3 |
| Glucose (mg/dL) | 96.4 ± 17.2 | 96.5 ± 14.8 | 94.0 ± 14.3 | 97.2 ± 17.9 |
| CRP (mg/L) | 0.6 ± 0.8 | 0.7 ± 1.2 | 0.6 ± 0.5 | 0.8 ± 1.1 |
| **Underlying diseases** |  |  |  |  |
| Hypertension | 4 (9.1) |  |  |  |
| Diabetes mellitus | 2 (4.5) |  |  |  |
| Dyslipidemia | 3 (6.8) |  |  |  |
| HBV carrier | 3 (6.8) |  |  |  |
| BMI, body mass index; WBC, white blood cell; ANC, absolute neutrophil count; MCV, mean corpuscular volume; MCH, mean corpuscular hemoglobin; MCHC, mean corpuscular hemoglobin concentration; BUN, blood urea nitrogen; HDL, high density lipoprotein; LDL, low density lipoprotein; AST, aspartate transaminase; ALT, alanine transaminase; GGT, gamma-glutamyl transferase; CRP, C-reactive protein; HBV, hepatitis B.  Continuous variables are shown as the median ± interquartile range, whereas categorical variables as numbers (percentage). | | | | |

| **Table S2. Comparison of characteristics with respect to the antibody half-life in the BNT162b2 cohort** | | | | |  |
| --- | --- | --- | --- | --- | --- |
| **Characteristics** | **Slow decay** (*n* = 12) |  | **Rapid decay** (*n* = 11) | ***p* value** | |
|  | **V1** |  | **V1** |  |  |
| **Age (years)** | 31.7 ± 6.7 |  | 33.5 ± 7.9 | 0.565 | |
| **Sex (women) (%)** | 8 (66.7%) |  | 9 (81.8%) | 0.725 | |
| **BMI (kg/m^2^)** | 22.4 ± 3.8 |  | 22.2 ± 4.2 | 0.904 | |
| **Anti-SARS-CoV-2 S IgG (U/mL)** | 123.9 ± 191.3 |  | 118.6 ± 56.6 | 0.928 | |
| **Antibody half-life** | 398.8 ± 462.0 |  | 98.3 ± 23.7 | 0.046 | |
| **Laboratory test results** |  |  |  |  | |
| WBC (10^3^/μL) | 6.1 ± 1.1 |  | 6.4 ± 1.7 | 0.643 | |
| ANC (/μL) | 13.7 ± 1.0 |  | 13.5 ± 1.4 | 0.757 | |
| Hemoglobin (g/dL) | 3286.3 ± 1025.6 |  | 3190.5 ± 1256.2 | 0.843 | |
| MCV (fL) | 90.0 ± 3.7 |  | 90.9 ± 4.5 | 0.619 | |
| MCH (pg) | 29.9 ± 1.4 |  | 30.0 ± 1.7 | 0.812 | |
| MCHC (g/dL) | 33.2 ± 0.6 |  | 33.0 ± 0.6 | 0.492 | |
| Platelet count (10^3^/μL) | 275.8 ± 89.1 |  | 271.5 ± 50.3 | 0.892 | |
| BUN (mg/dL) | 12.9 ± 3.7 |  | 11.4 ± 3.4 | 0.304 | |
| Creatinine (mg/dL) | 0.6 ± 0.1 |  | 0.6 ± 0.1 | 0.463 | |
| Albumin (g/dL) | 4.7 ± 0.2 |  | 4.7 ± 0.2 | 0.946 | |
| HDL cholesterol (mg/dL) | 62.8 ± 18.4 |  | 61.1 ± 13.6 | 0.810 | |
| LDL cholesterol (mg/dL) | 104.3 ± 26.1 |  | 112.8 ± 18.7 | 0.384 | |
| Triglyceride (mg/dL) | 114.8 ± 65.7 |  | 113.5 ± 65.8 | 0.960 | |
| AST (IU/L) | 28.4 ± 8.5 |  | 25.3 ± 10.2 | 0.429 | |
| ALT (IU/L) | 28.0 ± 22.2 |  | 33.4 ± 46.9 | 0.735 | |
| GGT (IU/L) | 22.2 ± 16.0 |  | 32.2 ± 50.6 | 0.545 | |
| Total bilirubin (mg/dL) | 0.8 ± 0.4 |  | 0.7 ± 0.3 | 0.521 | |
| Glucose (mg/dL) | 91.0 ± 11.3 |  | 93.2 ± 12.3 | 0.662 | |
| CRP (mg/L) | 0.6 ± 0.6 |  | 0.5 ± 0.5 | 0.644 | |
| **Underlying diseases** |  |  |  |  | |
| Hypertension | 1 (8.3) |  | 1 (9.1) | > 0.999 | |
| Diabetes mellitus | 1 (8.3) |  | 0 (0.0) | > 0.999 | |
| Dyslipidemia | 1 (8.3) |  | 1 (9.1) | > 0.999 | |
| HBV carrier | 0 (0.0) |  | 0 (0.0) | > 0.999 | |
| BMI, body mass index; WBC, white blood cell; ANC, absolute neutrophil count; MCV, mean corpuscular volume; MCH, mean corpuscular hemoglobin; MCHC, mean corpuscular hemoglobin concentration; BUN, blood urea nitrogen; HDL, high density lipoprotein; LDL, low density lipoprotein; AST, aspartate transaminase; ALT, alanine transaminase; GGT, gamma-glutamyl transferase; CRP, C-reactive protein; HBV, hepatitis B.  Continuous variables are shown as the median ± interquartile range, whereas categorical variables as numbers (percentage). | | | | |  |

| **Table S3. Comparison of characteristics with respect to the antibody half-life in the ChAdOx1 cohort** | | | | |  |  |
| --- | --- | --- | --- | --- | --- | --- |
| **Characteristics** | **Slow decay** (*n* = 11) |  | **Rapid decay** (*n* = 10) | ***p* value** | | |
|  | **V1** |  | **V1** |  |  |  |
| **Age (years)** | 39.8 ± 13.0 |  | 41.7 ± 10.0 | 0.716 | | |
| **Sex (women) (%)** | 9 (81.8%) |  | 7 (70.0%) | 0.903 | | |
| **BMI (kg/m^2^)** | 21.8 ± 3.0 |  | 24.6 ± 3.9 | 0.088 | | |
| **Anti-SARS-CoV-2 S IgG (U/mL)** | 398.8 ± 462.0 |  | 98.3 ± 23.7 | 0.046 | | |
| **Antibody half-life** | 97.9 ± 56.1 |  | 54.2 ± 8.6 | 0.036 | | |
| **Laboratory test results** |  |  |  |  | | |
| WBC (10^3^/μL) | 5.8 ± 1.6 |  | 5.8 ± 1.3 | 0.916 | | |
| ANC (/μL) | 3060.5 ± 777.1 |  | 3316.7 ± 1220.8 | 0.569 | | |
| Hemoglobin (g/dL) | 13.3 ± 0.7 |  | 13.7 ± 0.9 | 0.363 | | |
| MCV (fL) | 92.8 ± 5.8 |  | 90.6 ± 3.9 | 0.330 | | |
| MCH (pg) | 31.1 ± 2.4 |  | 30.2 ± 1.4 | 0.326 | | |
| MCHC (g/dL) | 33.5 ± 0.6 |  | 33.4 ± 0.4 | 0.600 | | |
| Platelet count (10^3^/μL) | 272.6 ± 45.6 |  | 269.9 ± 71.2 | 0.917 | | |
| BUN (mg/dL) | 11.0 ± 3.5 |  | 13.6 ± 3.6 | 0.110 | | |
| Creatinine (mg/dL) | 0.6 ± 0.1 |  | 0.7 ± 0.2 | 0.207 | | |
| Albumin (g/dL) | 4.5 ± 0.2 |  | 4.4 ± 0.1 | 0.706 | | |
| HDL cholesterol (mg/dL) | 71.5 ± 14.3 |  | 61.9 ± 11.0 | 0.105 | | |
| LDL cholesterol (mg/dL) | 108.6 ± 23.8 |  | 120.4 ± 35.7 | 0.381 | | |
| Triglyceride (mg/dL) | 75.3 ± 26.2 |  | 113.2 ± 62.9 | 0.102 | | |
| AST (IU/L) | 21.0 ± 4.3 |  | 21.5 ± 6.2 | 0.832 | | |
| ALT (IU/L) | 12.7 ± 4.9 |  | 21.7 ± 12.3 | 0.053 | | |
| GGT (IU/L) | 15.5 ± 6.5 |  | 17.0 ± 5.2 | 0.555 | | |
| Total bilirubin (mg/dL) | 0.7 ± 0.3 |  | 0.7 ± 0.2 | 0.448 | | |
| Glucose (mg/dL) | 102.6 ± 27.6 |  | 99.4 ± 11.7 | 0.728 | | |
| CRP (mg/L) | 0.3 ± 0.1 |  | 1.2 ± 1.4 | 0.085 | | |
| **Underlying diseases** |  |  |  |  | | |
| Hypertension | 1 (9.1) |  | 1 (10.0) | > 0.999 | | |
| Diabetes mellitus | 1 (9.1) |  | 0 (0.0) | > 0.999 | | |
| Dyslipidemia | 0 (0.0) |  | 1 (10.0) | 0.961 | | |
| HBV carrier | 1 (9.1) |  | 2 (20.0) | 0.929 | | |
| BMI, body mass index; WBC, white blood cell; ANC, absolute neutrophil count; MCV, mean corpuscular volume; MCH, mean corpuscular hemoglobin; MCHC, mean corpuscular hemoglobin concentration; BUN, blood urea nitrogen; HDL, high density lipoprotein; LDL, low density lipoprotein; AST, aspartate transaminase; ALT, alanine transaminase; GGT, gamma-glutamyl transferase; CRP, C-reactive protein; HBV, hepatitis B.  Continuous variables are shown as the median ± interquartile range, whereas categorical variables as numbers (percentage). | | | | | |  |

| **Table S4. Comparison of characteristics with respect to the fold increase in antibody titers following BNT162b2 booster vaccination** | | | | |  |
| --- | --- | --- | --- | --- | --- |
| **Characteristics** | **High responder** (*n* = 22) |  | **Low responder** (*n* = 22) | ***p* value** |  |
|  | **V1** |  | **V1** |  |  |
| **Age (years)** | 38.0 ± 10.8 |  | 34.9 ± 9.6 | 0.314 |  |
| **Sex (women) (%)** | 16 (72.7%) |  | 17 (77.3%) | > 0.999 |  |
| **BMI (kg/m^2^)** | 23.2 ± 4.0 |  | 22.2 ± 3.5 | 0.396 |  |
| **Anti-SARS-CoV-2 S IgG (U/mL)** | 82.7 ± 57.3 |  | 113.8 ± 149.7 | 0.371 |  |
| **Laboratory test results** |  |  |  |  |  |
| WBC (10^3^/μL) | 6.3 ± 1.7 |  | 5.8 ± 1.1 | 0.262 |  |
| ANC (/μL) | 3298.5 ± 1187.1 |  | 3127.1 ± 908.1 | 0.593 |  |
| Hemoglobin (g/dL) | 13.6 ± 1.1 |  | 13.5 ± 0.9 | 0.571 |  |
| MCV (fL) | 90.3 ± 5.0 |  | 91.8 ± 3.9 | 0.287 |  |
| MCH (pg) | 30.1 ± 2.0 |  | 30.5 ± 1.6 | 0.400 |  |
| MCHC (g/dL) | 33.3 ± 0.6 |  | 33.3 ± 0.5 | 0.980 |  |
| Platelet count (10^3^/μL) | 282.3 ± 58.8 |  | 262.9 ± 69.9 | 0.323 |  |
| BUN (mg/dL) | 11.8 ± 3.6 |  | 12.6 ± 3.7 | 0.493 |  |
| Creatinine (mg/dL) | 0.7 ± 0.2 |  | 0.6 ± 0.1 | 0.492 |  |
| Albumin (g/dL) | 4.5 ± 0.2 |  | 4.6 ± 0.2 | 0.528 |  |
| HDL cholesterol (mg/dL) | 62.1 ± 12.9 |  | 66.5 ± 16.6 | 0.336 |  |
| LDL cholesterol (mg/dL) | 114.8 ± 27.1 |  | 107.5 ± 25.6 | 0.365 |  |
| Triglyceride (mg/dL) | 107.4 ± 56.7 |  | 101.0 ± 60.7 | 0.721 |  |
| AST (IU/L) | 24.0 ± 8.4 |  | 24.4 ± 7.9 | 0.868 |  |
| ALT (IU/L) | 26.9 ± 34.1 |  | 21.3 ± 18.1 | 0.505 |  |
| GGT (IU/L) | 24.5 ± 36.0 |  | 19.2 ± 12.7 | 0.520 |  |
| Total bilirubin (mg/dL) | 0.8 ± 0.4 |  | 0.7 ± 0.2 | 0.396 |  |
| Glucose (mg/dL) | 99.0 ± 21.2 |  | 93.7 ± 12.0 | 0.317 |  |
| CRP (mg/L) | 0.6 ± 0.5 |  | 0.7 ± 1.1 | 0.696 |  |
| **Underlying diseases** |  |  |  |  |  |
| Hypertension | 2 (9.1) |  | 2 (9.1) | > 0.999 |  |
| Diabetes mellitus | 1 (4.5) |  | 1 (4.5) | > 0.999 |  |
| Dyslipidemia | 2 (9.1) |  | 1 (4.5) | > 0.999 |  |
| HBV carrier | 1 (4.5) |  | 2 (9.1) | > 0.999 |  |
| BMI, body mass index; WBC, white blood cell; ANC, absolute neutrophil count; MCV, mean corpuscular volume; MCH, mean corpuscular hemoglobin; MCHC, mean corpuscular hemoglobin concentration; BUN, blood urea nitrogen; HDL, high density lipoprotein; LDL, low density lipoprotein; AST, aspartate transaminase; ALT, alanine transaminase; GGT, gamma-glutamyl transferase; CRP, C-reactive protein; HBV, hepatitis B.  Continuous variables are shown as the median ± interquartile range, whereas categorical variables as numbers (percentage). | | | | | |

| **Table S5. Genus-level taxonomic markers linked to antibody durability following the two-dose primary vaccination series** | | | | | | | | |
| --- | --- | --- | --- | --- | --- | --- | --- | --- |
| **BNT162b2** | | | | | | | | |
| **Taxon name** | **Taxon rank** | | | **Taxonomy** | **LDA effect size** | | | ***p* value** |
| *Prevotella* | | Genus | Bacteria: Bacteroidetes: Bacteroidia: Bacteroidales: Prevotellaceae | | | 4.62838 | | 0.0147 |
| *Faecalibacterium* | | Genus | Bacteria: Firmicutes: Clostridia: Clostridiales: Ruminococcaceae | | | 4.26036 | | 0.04791 |
| *Subdoligranulum* | | Genus | Bacteria: Firmicutes: Clostridia: Clostridiales: Ruminococcaceae | | | 3.98239 | | 0.01467 |
| *Agathobacter* | | Genus | Bacteria: Firmicutes: Clostridia: Clostridiales: Lachnospiraceae | | | 3.50072 | | 0.04703 |
| *PAC000195_g* | | Genus | Bacteria: Firmicutes: Clostridia: Clostridiales: Lachnospiraceae | | | 3.13955 | | 0.0476 |
| *Eubacterium_g23* | | Genus | Bacteria: Firmicutes: Clostridia: Clostridiales: Ruminococcaceae | | | 3.05084 | | 0.00962 |
| *PAC000196_g* | | Genus | Bacteria: Firmicutes: Clostridia: Clostridiales: Lachnospiraceae | | | 2.64999 | | 0.02783 |
| *PAC000692_g* | | Genus | Bacteria: Firmicutes: Clostridia: Clostridiales: Lachnospiraceae | | | 2.54384 | | 0.01635 |
| *PAC001236_g* | | Genus | Bacteria: Firmicutes: Clostridia: Clostridiales: Mogibacterium_f | | | 2.10817 | | 0.00813 |
| **ChAdOx1** | | | | | | | | |
| **Taxon name** | **Taxon rank** | | | **Taxonomy** | **LDA effect size** | | | ***p* value** |
| *Alistipes* | | Genus | Bacteria: Bacteroidetes: Bacteroidia: Bacteroidales: Rikenellaceae | | | | 4.46179 | 0.03844 |
| *Escherichia* | | Genus | Bacteria: Proteobacteria: Gammaproteobacteria: Enterobacterales: Enterobacteriaceae | | | | 3.8579 | 0.00937 |
| *Parabacteroides* | | Genus | Bacteria: Bacteroidetes: Bacteroidia: Bacteroidales: Porphyromonadaceae | | | | 3.85644 | 0.01235 |
| *Enterococcus* | | Genus | Bacteria: Firmicutes: Bacilli: Lactobacillales: Enterococcaceae | | | | 3.23579 | 0.04323 |
| LDA, linear discriminant analysis. LDA effect size and *p* value are expressed as values of V2. | | | | | | | | |

| **Table S6. Functional markers linked to antibody durability following the two-dose primary vaccination series** | | | | | |
| --- | --- | --- | --- | --- | --- |
| **BNT162b2** | | | | | |
| **Ortholog** | **Definition** | **LDA effect size** | ***p* value** |  |  |
| K21572 | starch-binding outer membrane protein, SusD/RagB family | 3.027146747 | 0.029558584 |  |  |
| K03088 | RNA polymerase sigma-70 factor, ECF subfamily | 2.898153528 | 0.034856847 |  |  |
| K02014 | iron complex outer membrane receptor protein | 2.892464376 | 0.012222799 |  |  |
| K21573 | TonB-dependent starch-binding outer membrane protein SusC | 2.83328505 | 0.034856847 |  |  |
| K07133 | uncharacterized protein | 2.583616985 | 0.006862172 |  |  |
| K03327 | multidrug resistance protein, MATE family | 2.563599214 | 0.040945697 |  |  |
| K02005 | HlyD family secretion protein | 2.534941683 | 0.014699148 |  |  |
| K04763 | integrase/recombinase XerD | 2.51074145 | 0.047912755 |  |  |
| K03080 | unknown | 2.435667743 | 0.029558584 |  |  |
| K03496 | chromosome partitioning protein | 2.427397893 | 0.005615999 |  |  |
| K06921 | uncharacterized protein | 2.354119017 | 0.006862172 |  |  |
| K07114 | Ca-activated chloride channel homolog | 2.353981482 | 0.017607529 |  |  |
| K01209 | alpha-N-arabinofuranosidase | 2.327508772 | 0.034856847 |  |  |
| K03427 | type I restriction enzyme M protein | 2.261008694 | 0.024968249 |  |  |
| K12132 | eukaryotic-like serine/threonine-protein kinase | 2.23790451 | 0.024968249 |  |  |
| K07481 | transposase, IS5 family | 2.230807326 | 0.047912755 |  |  |
| K00934 | arginine kinase | 2.214747825 | 0.021008501 |  |  |
| K12373 | hexosaminidase | 2.21414556 | 0.034856847 |  |  |
| K00945 | CMP/dCMP kinase | 2.198550732 | 0.01012345 |  |  |
| K03497 | chromosome partitioning protein, ParB family | 2.193478514 | 0.040945697 |  |  |
| K07670 | two-component system, OmpR family, response regulator MtrA | 2.190620579 | 0.040945697 |  |  |
| K03086 | RNA polymerase primary sigma factor | 2.176113292 | 0.012222799 |  |  |
| K01153 | type I restriction enzyme, R subunit | 2.166736295 | 0.014699148 |  |  |
| K01206 | alpha-L-fucosidase | 2.155092232 | 0.047912755 |  |  |
| K03797 | carboxyl-terminal processing protease | 2.152596837 | 0.040945697 |  |  |
| K01972 | DNA ligase (NAD+) | 2.151232639 | 0.006862172 |  |  |
| K02316 | DNA primase | 2.149922687 | 0.012222799 |  |  |
| K01278 | dipeptidyl-peptidase 4 | 2.139006486 | 0.003716487 |  |  |
| K15923 | alpha-L-fucosidase 2 | 2.138334543 | 0.012222799 |  |  |
| K01897 | long-chain acyl-CoA synthetase | 2.134146748 | 0.006862172 |  |  |
| K02429 | MFS transporter, FHS family, L-fucose permease | 2.130616552 | 0.024968249 |  |  |
| K03654 | ATP-dependent DNA helicase RecQ | 2.0961909 | 0.029558584 |  |  |
| K06142 | outer membrane protein | 2.091672354 | 0.029558584 |  |  |
| K07668 | two-component system, OmpR family, response regulator VicR | 2.089531242 | 0.029558584 |  |  |
| K20444 | O-antigen biosynthesis protein | 2.087238457 | 0.040945697 |  |  |
| K03773 | FKBP-type peptidyl-prolyl cis-trans isomerase FklB | 2.084959015 | 0.021008501 |  |  |
| K01372 | bleomycin hydrolase | 2.083376629 | 0.047912755 |  |  |
| K00845 | glucokinase | 2.082767625 | 0.034856847 |  |  |
| K13993 | HSP20 family protein | 2.077882744 | 0.003005135 |  |  |
| K12340 | outer membrane protein | 2.075657284 | 0.047912755 |  |  |
| K05989 | alpha-L-rhamnosidase | 2.074244914 | 0.040945697 |  |  |
| K09955 | uncharacterized protein | 2.046335704 | 0.047912755 |  |  |
| K01740 | O-acetylhomoserine (thiol)-lyase | 2.046256083 | 0.021008501 |  |  |
| K03308 | neurotransmitter:Na+ symporter, NSS family | 2.046220228 | 0.034856847 |  |  |
| K07011 | uncharacterized protein | 2.041013115 | 0.01012345 |  |  |
| K00705 | 4-alpha-glucanotransferase | 2.037227266 | 0.004577751 |  |  |
| K02520 | translation initiation factor IF-3 | 2.031427673 | 0.024968249 |  |  |
| K03406 | methyl-accepting chemotaxis protein | 2.022128561 | 0.029558584 |  |  |
| K04717 | sphingosine-1-phosphate phosphotase 2 | 2.004900073 | 0.024968249 |  |  |
| K01915 | glutamine synthetase | 2.000276167 | 0.021008501 |  |  |
| **Module (PICRUSt)** | **Definition** | **LDA effect size** | ***p* value** |  |  |
| M00157 | F-type ATPase, prokaryotes and chloroplasts | 2.934897197 | 0.006862172 |  |  |
| M00620 | Incomplete reductive citrate cycle, acetyl-CoA => oxoglutarate | 2.699789877 | 0.040945697 |  |  |
| **Module (MinPath)** | **Definition** | **LDA effect size** | ***p* value** |  |  |
| M00030 | Lysine biosynthesis, AAA pathway, 2-oxoglutarate => 2-aminoadipate => lysine | 2.742624691 | 0.014699148 |  |  |
| M00346 | Formaldehyde assimilation, serine pathway | 2.731849608 | 0.017607529 |  |  |
| **Pathway (PICRUSt)** | **Definition** | **LDA effect size** | ***p* value** |  |  |
| ko04142 | Lysosome | 2.539337125 | 0.029558584 |  |  |
| ko00195 | Photosynthesis | 2.466708031 | 0.008351423 |  |  |
| **Pathway (MinPath)** | **Definition** | **LDA effect size** | ***p* value** |  |  |
| ko00750 | Vitamin B6 metabolism | 3.159378505 | 0.013823024 |  |  |
| ko00540 | Lipopolysaccharide biosynthesis | 3.094327283 | 0.011623419 |  |  |
| **ChAdOx1** | | | |  |  |
| **Ortholog** | **Definition** | **LDA effect size** | ***p* value** |  |  |
| K07480 | insertion element IS1 protein InsB | 2.475728734 | 0.009374768 |  |  |
| K07483 | transposase | 2.443954282 | 0.009374768 |  |  |
| K01155 | type II restriction enzyme | 2.164906398 | 0.0028546 |  |  |
| K18138 | multidrug efflux pump | 2.13039138 | 0.034264008 |  |  |
| K01607 | 4-carboxymuconolactone decarboxylase | 2.013597303 | 0.043308143 |  |  |
| **Module (PICRUSt)** | **Definition** | **LDA effect size** | ***p* value** |  |  |
| M00064 | ADP-L-glycero-D-manno-heptose biosynthesis | 2.50395649 | 0.016144778 |  |  |
| M00532 | Photorespiration | 2.401161492 | 0.043308143 |  |  |
| **Module (MinPath)** | **Definition** | **LDA effect size** | ***p* value** |  |  |
| M00565 | Trehalose biosynthesis, D-glucose 1P => trehalose | 2.708205975 | 0.009374768 |  |  |
| **Pathway (PICRUSt)** | **Definition** | **LDA effect size** | ***p* value** |  |  |
| - | - | - | - |  |  |
| **Pathway (MinPath)** | **Definition** | **LDA effect size** | ***p* value** |  |  |
| - | - | - | - |  |  |
| LDA, linear discriminant analysis. LDA effect size and *p* value are expressed as values of V2. | | | |  |  |

| **Table S7. Taxonomic markers at the genus level associated with the high fold increase in antibody titers following BNT162b2 booster vaccination** | | | | | | | |
| --- | --- | --- | --- | --- | --- | --- | --- |
| **Taxon name** | **Taxon rank** | | | **Taxonomy** | **LDA effect size** | | ***p* value** |
| *Faecalibacterium* | | Genus | Bacteria: Firmicutes: Clostridia: Clostridiales: Ruminococcaceae | | | 4.40882 | 0.03998 |
| *Clostridium* | | Genus | Bacteria: Firmicutes: Clostridia: Clostridiales: Lachnospiraceae | | | 3.65726 | 0.02734 |
| *Agathobaculum* | | Genus | Bacteria: Firmicutes: Clostridia: Clostridiales: Ruminococcaceae | | | 2.41073 | 0.03071 |
| *JRNA_g* | | Genus | Bacteria: Firmicutes: Clostridia: Clostridiales: Mogibacterium_f | | | 2.31663 | 0.04727 |
| *PAC000672_g* | | Genus | Bacteria: Firmicutes: Clostridia: Clostridiales: Ruminococcaceae | | | 2.20235 | 0.01615 |
| LDA, linear discriminant analysis. LDA effect size and *p* value are expressed as values of V3. | | | | | | | |

**Supplementary References**

1. Beghini F, McIver LJ, Blanco-Míguez A, Dubois L, Asnicar F, Maharjan S, et al. Integrating taxonomic, functional, and strain-level profiling of diverse microbial communities with bioBakery 3. *eLife.* 2021;10:e65088.

2. Anderson, M. J. A new method for non-parametric multivariate analysis of variance. *Austral Ecology.* 2001;26:32-46.
